# What happens after stopping IN Esketamine? A prospective report of case series

**DOI:** 10.1192/j.eurpsy.2026.11426

**Published:** 2026-07-31

**Authors:** T. Sagues, A. Barnés, C. Fernández, D. Vegas, G. Fucho, V. Gálvez

**Affiliations:** Consorci Sanitari Parc Taulí, Sabadell, Spain

## Abstract

**Introduction:**

Intranasal esketamine has emerged as an innovative option for the management of treatment-resistant depression (TRD), offering a novel mechanism of action and rapid antidepressant effects. However, evidence regarding the clinical trajectory after treatment discontinuation remains limited, raising questions about the durability of its therapeutic benefits and the optimal maintenance strategies.

**Objectives:**

The primary objective of this study was to evaluate the clinical course of patients with treatment-resistant depression (TRD) after discontinuation of intranasal esketamine, assessing the durability of antidepressant response and remission status over a four-month follow-up period.

A secondary objective was to explore whether sustained remission could be maintained without ongoing esketamine treatment following an adequate acute and maintenance course.

**Methods:**

We conducted a prospective observational study involving outpatients diagnosed with TRD. Six patients received intranasal esketamine, and one received subcutaneous racemic ketamine. Sociodemographic, clinical, treatment-related, and outcome data were collected. Depression severity was assessed using the Montgomery–Åsberg Depression Rating Scale (MADRS) at baseline, at the end of treatment, and monthly for four months following treatment discontinuation. Remission was defined as a MADRS score ≤10. Treatment resistance was defined as nonresponse to at least three antidepressant strategies during the current depressive episode.

**Results:**

Seven patients were included (5 women and 2 men; mean age: 59 years). All were diagnosed with recurrent TRD, except one case of single-episode major depressive disorder. Additionally, two patients exhibited resistance to electroconvulsive therapy (ECT) in the current episode. The mean MADRS score at the end of treatment was 2.7, and all patients (100%) achieved clinical remission. Four months after discontinuing esketamine, all patients (100%) remained in remission.

**Conclusions:**

These findings suggest that discontinuation of esketamine after an adequate acute and maintenance treatment course may be feasible in certain patient profiles, with sustained clinical benefits. For patients achieving remission at treatment completion, remission may persist following discontinuation of esketamine.

**Disclosure of Interest:**

None Declared

